# Mindfulness, Buddhist Modernity and Cultural Psychology

**DOI:** 10.1007/s12124-024-09852-w

**Published:** 2024-06-06

**Authors:** Bo Allesøe Christensen

**Affiliations:** Aalborg University, Aalborg, Denmark

**Keywords:** Mindfulness, Buddhist modernity, Context sensitivity, De-parochialism

## Abstract

I will in this article use Fircks as a point of departure for trying to understand the complexities involved in the use of the concept of mindfulness. As will be seen, mindfulness can be traced to a decoupling from a religious background and subsequent appropriation within several Western contexts. This will then be used for a discussion of how to deal with the historicity of the phenomena studied within cultural psychology. Here two reminders will be suggested, namely understanding phenomena through a context-sensibility and at the same time being aware of any disciplinary parochialism.

## Introduction

Fircks (2023) aims at presenting a different concept of mindfulness by using his own experience practicing mindfulness. Being inspired by Herman Hesse and Alan Watts’ works with Buddhism and Taoism, he interprets this from a Meadian perspective. In this brief comment on the article, I will raise one specific reservation regarding parts of the approach taken. I will, first, pick up one of Fircks’ good ideas – that we should pay more attention to (or be more mindfull of!) the historical contextual features of what we are investigating, thereby creating a better foundation for developing theories. My reservation will consist of two parts. The first will emphasize that drawing on a specific western understanding of mindfulness might actually be an example of not paying enough attention to the historical features of the phenomena studied. In a cultural psychological sense, we might even speak of a sort of cultural appropriation, what in *religionswissenschaft* is called the Buddhist modernity. Though Fircks’ point of departure is Taoism and not Buddhism *per se*, the notion of Buddhist modernity is used as an overall expression of how the Western part of the world has appropriated concepts and practices – like meditation – from eastern religions, creating what Said (1978) called a cultural representation(s) of the Orient. Furthermore, many scholars agree that though Buddhism and Taoism are two different religions originating in two different countries, the exchange of ideas and practices between them, especially meditation practices, makes it difficult to establish what practice belongs originally to what. Hence, many scholars of religion maintain that most if not all religions have some sort of syncretistic foundation, made up of exchanges from other religions or religious practices, and often incorporating new practices, ideas and interpretations as religions historically spread to new geographical areas. My reservation will therefore, second, also serve as a brief cultural psychological reminder, that contextualizing phenomena, as emphasized by Fircks, is elucidating historical conditions not only of the phenomena studied but also of how we choose to approach or investigate them. So, I will start by bringing out the part from Fircks’ article I will focus on, then present my reservation and last the reminder.

## Fircks – Mindfulness and Taoism

As Fircks starts out noticing, studies in mindfulness have increased over the last decades creating a vast amount of data evaluating the effect of (techniques of) mindfulness based a number of factors we as a society and as individuals consider valuable, like peace of mind, lowering states of anxiety, better skills of concentration etc. The trouble as Fircks (2023) claims, is that

“…most of the current mindfulness-based studies treat mindfulness solely as a variable that can be analyzed with statistical instruments. By the positivistic imperative of their studies, these authors create manuscripts that are essentially a-theoretical and ahistorical” (page 2).

Similar to recent work arguing against a psychological focus on variables (Valsiner, 2023) Firck’s suggestion is, that by focusing on the historical conditions of mindfulness we will become aware of the complexities and varieties of how it is practiced, and thereby gain in overall understanding and theoretical potency. As Fircks’ put it “I argue that mindfulness-based research can be stimulated if we include historical and theoretical features in our academic inquiry” (ibid.)

His approach to this theoretical and historical enlargement of mindfulness in psychological research, is combining what he terms historical milestones of Taoism with what he claims is G. H. Meads cultural-psychological framework. His aim thereby is showing what happens during the practice of mindfulness by drawing on his own autoethnographic material. As already indicated my reservations relates to the historical background he claims to incorporate, mainly because this historical background is somewhat myopic. It is appropriated mainly from people contributing to the creation of a specific western conception of the Orient and of mindfulness, and not a cultural psychological or based on scholars specializing in understanding the cultural contexts and historical development of the concept of mindfulness. In Fircks case, Herman Hesse, Alan Watts and C. G. Jung are used as key interpreters of Taoism and Buddhism, and hence as providing a specific frame for understanding mindfulness, authors significant in contributing to what McMahan terms Buddhist modernism.

## The Orientalist Myth

Any scholar trying to understand eastern religions (or any other religion for that matter) will have to pay close attention to how these religions have been presented historically in the western hemisphere since the era of colonization. Said (1978) was one of the first to emphasize, that western representations of the Orient are implicitly tied to the ideologies and political programs of the selfsame west trying to subjugate people of the middle east as well as Asia. At first, this was of course a matter of claiming land, but later it had more to do with subsuming, in the following case, an Asian worldview under a western mode of interpretation. Let me use Inden’s studies of India (1986; 1990) as a first example. He invokes Collingwood claiming that any social science will lapse into a spurious essentialism, if the agent of history is taken to be an ideal or a material ‘something’ that underlies and affects but is not itself affected by historical acts (Inden, 1990, 18). In the case of India, as Inden shows, the dominant colonial Western interpretation deprived Indians and their practices of their own agency and rationality, understanding these through various essences, like caste, Hinduism, the Indian mind, etc., instead. Let us use caste as an example.

According to Inden’s interpretation there has historically been an over-emphasis on caste by Western interpretations of India to such an extent that,

“Caste, then, is assumed to be the essence of Indian civilization. People in India are not even partially autonomous agents. They do not shape and reshape their world. Rather they are the patients of that which makes them Indians–the social, material reality of caste. The people of India are not the makers of their own history. A hidden, substantialized Agent, Caste, is the maker of it.” (1986, 428).

Among the many conditions contributing to this, and I am simplifying Inden’s argument here, was the British Imperialists’ effort of installing an order in what was otherwise conceived as a diverse and immeasurable Indian population. Successive censuses done by the British showed a bewildering variety of responses in terms of how and to what the Indian population saw themselves as affiliated. Hence, using a simplified notion of caste for pigeonholing the Indian population was seen as necessary corollary of imposing a unified political order (Inden, 1990). Thus, the essentialization of caste, Inden suggests, had a very pragmatic and imperialistic origin in the British effort of exercising control and power over the Indian population. Based on his study of Indian history, Inden therefore rejects the hitherto prevalent Western orientalist construction of Indian society as characterized essentially by caste from the inception of the Indian civilization down to the present. Inden claims instead that the idea of caste doesn’t appear until the thirteenth of fourteenth century (Inden, 1990, p. 82). Hence, the point is not that caste did not exist, it did. The point is, that it was used differently by the Indian population compared to how it was used by the British. A worry here could be, that the same or similar ‘thing’ has happened to the concept of mindfulness – that is has been essentialized and abstracted from the different contexts in which it has been used. To anticipate a section below, the concept (and not the concept of meditation!) is probably no more than 150 years of age but is often interpreted as providing an authentic possibility of self-healing distilled from an authentic essence of mediative practice within Buddhism and Taoism.

## Cultural Psychological Interlude

Inden can be said to follow Said in claiming that three characteristics defined the Orientalist discourse, and these constituted obstacles for understanding religious and other practices on their own terms:

“First, it is about the ‘civilized’ rather than about the ‘primitive’. This distinguishes it from anthropology which concerns itself with the latter more than the former. Second, it speaks of Asian Others in ways that contrast rather sharply with the way in which it speaks of itself. Third, it continually distinguishes the parts of Asia by reference to the same differentiating essences.” (1990, 37).

Furthermore, these characteristics served not just the colonial powers creating an Orientalist Other different from themselves. In the beginning it came to provide some of the ideological background of the establishment of academic studies investigating the Orient, eventually resulting in discussions like the rationality debates (Wilson, 1970), or how to dispense with the ideological leftover of colonization in the sciences.

Following Much (1995, 97–99) it can be claimed that overcoming similar obstacles have been the impetus behind developing a cultural psychology dealing with the possible bias of ethnocentrism and disciplinary parochialism. The ethnocentric part is, like the cahracteristics of the Orientalist discourse, the tendency to use norms and values from a western culture to judge and (d)evaluate the psychology of other cultures (e.g. understanding western countries as rationally and Asian countries as emotionally oriented, which, as a distinction, obviously makes sense only from a rational western point of view). By disciplinary parochialism is meant the use of models and categories based upon mostly experimental research within limited segments of Euro-American populations (i.e. that the western psychological discipline is WEIRD) as general models for what psychology is or should be. A genuine cultural psychology would, according to Much, instead “…base its categories, discriminations and generalizations upon empirical knowledge of the fullest possible range of existing human forms of life, without privileging one form as the norm or standard of evaluation” (1995, 99) Hence, similar to the concept of caste, we should be wary of essentializing an idea, concept or phenomena by abstracting from the varying contexts wherein it is used, let us call this a criteria of contextualization. But at the same time, we should in general be wary of the thoughts and ideas we presuppose about the phenomena investigated, avoiding these becoming obstacles to our understanding. Let us name this a criterion of de-parochialism (not to be confused with non-parochialism).

The interesting question is whether some of the thoughts presented above are relatable to our understanding of mindfulness? As we will see now, some of the same considerations has been presented when it comes to the idea of mindfulness.

## McMahan, Modernism and Buddhism

McMahan has in many ways done for Buddhism what Inden did for the case of India. He agrees with Said but ups the ante as we will see. Consider this description of Buddhism:

“Buddhism is a religion in which you don’t really have to believe anything in particular or follow any strict rules; you simply exercise compassion and maintain a peaceful state of mind through meditation. Buddhism values creativity and intuition and is basically compatible with a modern, scientific worldview. It is democratic, encourages freedom of thought, and is more of a “spirituality” than a religion.” (McMahan, 2008, p. 4).

As McMahan claims this is probably a description which most people would or could agree to. And rightly so. Because despite being very different and distinct from the Buddhism that have existed in Asia for centuries, McMahan claims that we are witnessing a new form of specific western Buddhism as a result of modernization, westernization, reinterpretation and reform taking place over the last century. This could be termed a domesticated Buddhism since it is stripped of mythological elements and superstitious cultural accretions, i.e. with fewer rituals, deemphasizing the miracles and supernatural events described in Buddhist literature, and stressing compatibility with scientific, humanistic and democratic ideals (McMahan, 2008, p. 5). As such this kind of modernist Buddhism is a little different from or probably more correct broader than the picture of orientalism that Said presented: “Orientalism has undoubtedly played a significant role in the creation of Buddhist modernism, but it would be mistaken to reduce all of Buddhist modernism to enactments of orientalist fantasies or to responses to colonialism or postcolonialism.” (McMahan, 2008, p. 20). The difference being that partakers in the construction of this kind of Buddhism has involved Asian Buddhists well acquainted with the western traditions as well as popular and semi-scholarly western authors, and sometimes both of them in a collaborative effort (McMahan mentions here the authors which Fircks bases his understanding on, namely Herman Hesse and Alan Watts, 2008, 195f, 228ff).

According to McMahan then, we face today not only one but many kinds of Buddhisms. Some with a focus on mediation, some with less focus on mediation and more about daily practices. Some are still very much influenced by specific geographical local practices and histories, while others take on a more hybrid character merging different kinds of cultural practices, like the modernist Buddhism we often associate with Buddhism in the west. If this is so, then a myriad of different understandings of mediation and meditative techniques are a reality as well, all with their different historical backgrounds. Which brings us back to Fircks’ demand of paying more attention to the historical background of mindfulness – including Fircks’ own version. Or, as indicated above, understanding mindfulness in a contextual and de-parochialised sense.

## Contextualizing Mindfulness

Obviously, this is a complex and ramified issue, so I will only indicate a couple of matters which is relevant to incorporate in the discussion of mindfulness addressed here. So, the basic worry here is, that Fircks in a certain sense suffers from the same problem he sees in previous research: namely, creating an ahistorical representation of mindfulness^1^. Hence, I will try to use the already mentioned criteria and point towards three issues to be reckoned with in terms of contextualizing mindfulness and remind ourselves of the diversity involved in understanding it as a concept. As a departure, I will use McMahan and Braun (2017) and Nielsen (2021) both exploring the historical background and contemporary use of the concept of mindfulness.

First of all, McMahan and Braun (2017) have pointed towards several contextual features of mindfulness, making it a more recent phenomena than one would imagine. What we see today is the recasting of meditation in scientific and pragmatic terms, framed within questions of effectiveness (in living), clinical applications and understandings of neurological activity, all implying this as a unique moment in the history of meditative practices developed from Indian and China. A significant part of this history is disconnecting the practice of mediation from its religious background, making it a secular practice not confined to monks but intended for the masses.

“In the disembedding of meditation from the monastery to the hospital, the health club, the company multipurpose room, and the military training center— as well as countless individuals’ bedrooms and meditation nooks— the practices themselves change, sometimes considerably, in order to fit into these new cultural, social, and institutional contexts.” (McMahan & Braun, 2017, p. 5).

In their genealogy tracing out some of the history of mindfulness, they start with a devastating fire burning down a huge part of the Burmese capital, Mandalay, in 1883. This saw a young monk, Ledi Sayadaw, taking off into the wild as he watched his monastery burn down, and at the same time fearing the demise of Buddhism. When the British took over Burma a couple of years later, Ledi “…saw the prospect of oblivion only grow larger and so began to promote meditation for all, even to the laity— even to women— as a means to preserve Buddhism.” (McMahan & Braun, 2017, p. 5).

This, according to McMahan and Braun, was the beginning of the mass practice of meditation not among the monks but within the laity, and this spread to other countries and eventually around the world as a secularized, standardized and widely practiced method. The effort set in motion by Sayadaw indicated that what was needed was not the deep meditative efforts requiring extended periods of retreat from the regular world, but certain insight techniques “…that required only a minimal level of concentration (a level called “momentary concentration” or khaṇikasamādhi) could be used to develop insight” (2017, 6). According to McMahan and Braun it was this streamlined meditation suited to laypeople which, combined with a growing popularity of meditation practices stemming from other religious traditions (e.g. Zen and Taoism), that spread to Western culture. The word mindfulness itself is, furthermore, a translation by the British scholar T. W. Rhys-Davis of the in Buddhism used Pali word *sati* connoting something like ‘bare attention’. However, with the translation into English followed the attachment of other meanings connoted by mindfulness like remembering, being thoughtful or being conscious of something (McMahan & Braun, 2017, p. 10; Nielsen, 2021, p. 52). Thus, through the translation mindfulness came also to signify a certain technique for establishing an attentive awareness in the individual.

Second, as Nielsen and others have pointed to, this individualization resonated in the European and American parts of the world due to how it was assimilated with romantic thinking. The idea of individuals having the potential *by themselves* to transform their own life, was an idea already developed both by Rousseau and the German romantics and the American transcendentalists, Emerson and Thoreau (Nielsen, 2021, p. 54) It is the same idea of individual freedom influencing the countercultures of the 50s and 60s stressing the reactionary and oppressive nature of the ‘stiff’ western societal orders, and hence the necessity of enabling individuals to practice a freedom of realizing or transforming themselves (Jay, 2006). To this end, people were drawn towards non-western societies appropriating practices like meditation including mindfulness, and following McMahan and Braun, the authors Fricks draws upon, Watts and Hesse, can be seen belonging to these counter cultures, albeit each in separate ways (2017, p. 9).

Furthermore, the individualization also meant that besides the romantic conception, mindfulness could be associated with both an evidence-based medical discourse, connected with perhaps the best known practitioner of mindfulness in medical practice, namely Kabat-Zinn (e.g. Kabat-Zinn, 1990). And at the same time also annexed within a neoliberal economical discourse by being turned into a commodity for the individual to purchase – what has been called Mcmindfulness (Nielsen, 2021, p. 53–56). According to Nielsen the last part has an important consequence, namely that:

“…mindfulness has become a commodity that has accelerated the striving to optimize performance; hence, mindfulness seems to have accelerated competition more than the opposite. In that sense, it has improved factors that enhance stress more than it has the opposite. A severe side effect of mindfulness as a cultural phenomenon is that produces the conditions that it is concurrently fighting.” (2021, p. 56).

So, contextualizing the use of the concept of mindfulness this way, we get a glimpse or an indication of the diversity of relations it has been used in and contributed to: a decoupling from Buddhism, a technique for the masses, an etymological expansion, supporting a romantic western conception of the individual, and finally as a commodity in the self-help industry.

Third, and briefly, the contextualization thus helps us be clearer about our own parochialism. As scholars we adopt perspectives and theories which appeal to us for various reasons. These come with a history as well, providing conceptual conditions influencing how we understand the phenomena we study. Thus, in Fircks’ case drawing on Herman Hesse, Alan Watts and using autoethnography implicitly impose a certain romantic, individualised understanding of using mindfulness as a concept. Now, there is nothing wrong with that, especially when elucidating specific uses and hence specific contextual understandings of the concept of mindfulness. The trouble starts, hopefully indicated through some of the examples above, when a specific use of the concept is elevated to the primary use.

## Conclusion

I have tried very briefly to indicate a reservation I have about Fircks article, namely that the position he adopts is not as historical committed as he wants scholars to be. Now, the departure here has only been one article, so my reservation might very well turn out to be unjustified considering other work he has done. Nevertheless, the discussion can still serve as a reminder of the two criteria mentioned above as part of doing cultural psychology. The context-sensibility implies that phenomena we try to understand must be understood *internally* to the context in which it occurs. In our case internally to the development and displacement of the concept of mindfulness between contexts ranging from Burma to America, and from lay-culture to counter-culture, and as related to other concepts like democratization, attention, rebelling, medicine, stress etc. Though perhaps only obvious on a superficial level, the intention was thereby to indicate a complex relationship of similarity and difference between the conceptual uses of mindfulness betwixt and between these contexts as a significant part of understanding the use of the concept of mindfulness as situated and historical. If one therefore elevates one aspect or feature of the concept to the detriment of the others, then it comes close to an act of essentializing, making the conceptual understanding ahistorical and external to any given context. If we succeed in not essentializing our conceptual understanding, then we implicitly recognize the historical situated (parochial) character of our investigation or analysis. But recognizing this has at the same time a de-parochializing effect because it establishes a bulwark against essentializing tendencies towards a-historicity.

*No data was used for this article, the author received no funding for conducting this study and has no competing interests to declare relevant to the content of this article.*

## Data Availability

No datasets were generated or analysed during the current study.

## Footnotes

1 He is not the only one. Another recent article published in IPBS by Lundh (2024) discusses the relationship between phenomenology and mindfulness, disregarding, for example, authors like Stone and Zahavi (2021) claiming that this often assumes a misleading presentation of phenomenology as a kind of meditative technique or practice. See also Sharf (1995) claiming that the privileging of meditative experience is a recent Buddhist modernist phenomena, which will be elaborated below.  
